# The Effects of Random Stimulation Rate on Measurements of Auditory Brainstem Response

**DOI:** 10.3389/fnhum.2020.00078

**Published:** 2020-03-20

**Authors:** Xin Wang, Mingxing Zhu, Oluwarotimi Williams Samuel, Xiaochen Wang, Haoshi Zhang, Junjie Yao, Yun Lu, Mingjiang Wang, Subhas Chandra Mukhopadhyay, Wanqing Wu, Shixiong Chen, Guanglin Li

**Affiliations:** ^1^CAS Key Laboratory of Human–Machine Intelligence-Synergy Systems, Shenzhen Institutes of Advanced Technology, Chinese Academy of Sciences (CAS), Shenzhen, China; ^2^Shenzhen College of Advanced Technology, University of Chinese Academy of Sciences, Shenzhen, China; ^3^The Duke Institute for Brain Sciences, Duke University, Durham, NC, United States; ^4^The School of Electronics and Information Engineering, Shenzhen Graduate School, Harbin Institute of Technology, Shenzhen, China; ^5^The Department of Engineering, Macquarie University, Sydney, NSW, Australia; ^6^The School of Biomedical Engineering, Sun Yat-Sen University, Guangzhou, China

**Keywords:** auditory brainstem response, random stimulation rate, hearing loss, hearing impairment, electroencephalogram

## Abstract

Electroencephalography (EEG) signal is an electrophysiological recording from electrodes placed on the scalp to reflect the electrical activities of the brain. Auditory brainstem response (ABR) is one type of EEG signals in response to an auditory stimulus, and it has been widely used to evaluate the potential disorders of the auditory function within the brain. Currently, the ABR measurements in the clinic usually adopt a fixed stimulation rate (FSR) technique in which the late evoked response could contaminate the ABR signals and deteriorate the waveform differentiation after averaging, thus compromising the overall auditory function assessment task. To resolve this issue, this study proposed a random stimulation rate (RSR) method by integrating a random interval between two adjacent stimuli. The results showed that the proposed RSR method was consistently repeatable and reliable in multiple trials of repeated measurements, and there was a large amplitude of successive late evoked response that would contaminate the ABR signals for conventional FSR methods. The ABR waveforms of the RSR method showed better wave I–V morphology across different stimulation rates and stimulus levels, and the improved ABR morphology played an important role in early diagnoses of auditory pathway abnormities. The correlation coefficients as functions of averaging time showed that the ABR waveform of the RSR method stabilizes significantly faster, and therefore, it could be used to speed up current ABR measurements with more reliable testing results. The study suggests that the proposed method would potentially aid the adequate reconstruction of ABR signals towards a more effective means of hearing loss screening, brain function diagnoses, and potential brain–computer interface.

## Introduction

Hearing impairment or hearing loss primarily occurs as a result of damage to a specific part of the ear due to congenital defects, diseases, exposure to excessively loud noise, or injury, among others. This phenomenon often leads to a decrease in the auditory sensitivity or hearing dysfunction that prevents humans from sensing sounds in their environment.

Individuals suffering from hearing impairment usually have difficulties in adequately perceiving and understanding what is spoken around them. Depending on where the damage occurs, hearing loss can be classified into different types that include conductive (outer-ear or middle-ear problem), sensory (inner-ear problem), neurological (auditory center problem), and mixed hearing loss (Elzouki et al., 2012). From recent studies, it was reported that hearing loss affects over 1.1 billion individuals across different age groups (World Health Organization, 2011; Olusanya et al., 2014; Vos et al., 2015). It leads to disability in about 50% (360–538 million) of the hearing loss populace with around 124 million persons having moderate to severe disability (World Health Organization, 2015). Meanwhile, it has been projected that the number of persons with hearing loss will continually increase with time since the number of affected individuals rose from 1.1 billion in 2013 to 1.4 billion in 2017 (James et al., 2018). Due to the growing number of patients, more and more attention are being paid to medical technology research. Some researchers pay attention to the construction of a physiological system platform and the development of some acquisition methods (Samuel et al., 2017a,b; Pirbhulal et al., 2018; Wu et al., 2018; de Oliveira et al., 2019). However, researches on the system or platform cannot solve the essential problems in clinically used technology and help less with the early detection in an auditory aspect. Therefore, there is an urgent need to develop an efficient strategy for early detection and timely treatment of hearing loss, to prevent the language development impediment of newborns and life quality decline of adults.

In clinical settings, the commonly used method for auditory function assessment is the auditory brainstem response (ABR) measurement, in which electrodes are placed on the scalp to record electrical brain activities in response to brief sound stimulation to the ear. Fundamentally, the ABR signal usually occurs within the first 10 ms following the stimulus onset, and it could reflect the functional status of the auditory pathway when the neural information of the incoming sound propagates from the auditory nerve to the auditory cortex. Therefore, the ABR measurement is commonly regarded as an objective method for evaluating the perceiving sensitivity of the auditory system (Galambos and Hecox, 1978; Avan and Bonfils, 1997; Alwan, 2012; Xie et al., 2018). ABR parameters such as the amplitude of peaks, the latency of waves I–V, interpeak latency, and interaural latency are very important for the detection of brainstem impairments and central auditory abnormities. For example, the absolute latency of wave I is prolonged, but interpeak latencies are not affected for conductive hearing loss. In contrast, wave I tends to be normal, but the interpeak latencies of waves I–III and I–IV are usually prolonged for neural hearing loss.

The lowest intensity at which wave V of ABR signals can be reliably observed could provide an estimation of the hearing threshold. However, it is still controversial to use the ABR thresholds as a replacement of the behavior pure-tone audiometry (PTA). For instance, Canale et al. (2020) reported that the mean difference between the ABR and PTA thresholds was about 20 dB in normal hearing, and no differences were found in conductive or sensorineural hearing loss. Ceylan et al. (2018) showed that the mean threshold difference was 5 dB at 1 kHz and that there was no significant difference at high frequencies. Lu et al. (2017) reported that the click-evoked ABR thresholds and PTA thresholds differed by less than 20 dB in 72.6% subjects at frequencies of 2–4 kHz. Hoda et al. (2019) showed that there was a high degree of correlation between click ABR and behavioral PTA thresholds. However, Talaat et al. (2020) claimed that the click- and tone burst-evoked ABR hearing thresholds significantly overestimate the behavioral threshold.

Among all the factors that may lead to the controversies of comparison between ABR and PTA thresholds, the most important factor might be that it is still a great challenge to obtain reliable ABR waveform morphologies for accurate diagnostic purposes, given that the ABR signal is rather low in amplitude (as low as 0.1 μV). For instance, the current method that adopts an overlapping averaging technique with a fixed stimulation rate (FSR) usually generates a relatively poor ABR waveform when the stimulation rate is high. Although the irrelevant noises could be attenuated during the averaging, the obtained ABR signals by the FSR method not only contain the target signals but also include evoked potential trails from the previous segments. The auditory evoked potential (AEP) is segmented in three parts, namely, according to different latencies: short latency response (SLR; 0–10 ms), middle latency response (MLR; 10–50 ms), and long latency response (LLR; >50 ms). The first part SLR is characterized by a lower amplitude, which is filtered to eliminate the power frequency and other environmental interferences and averaged *via* the overlapping technique to obtain the actual ABR signal (Wong and Bickford, 1980; Aimoni et al., 2010; Rouillon et al., 2016; Jiang et al., 2018). It should be noted that the temporal gap between two adjacent stimuli onset could be short for high stimulation rate, so the unwanted components from the previous late evoked response (MLR and LLR) would mix with the ABR signal of the current stimulation. For the FSR method, the interferences from the previous MLR and LLR components would be enhanced after fixed-rate averaging, leading to undesired ABR morphology alteration.

To reduce the interferences from the MLR and LLR components, Alvarez used an iterative randomized stimulation and averaging (iterative-RSA) and deconvolution method to measure ABR at a high stimulation rate (Alvarez et al., 2010; Valderrama et al., 2012, 2014). Their method could help to reduce the late response interferences through an iterative process in the time domain. However, the deconvolution algorithms (such as least-squares deconvolution and continuous-loop averaging deconvolution) involve complex computations which are rather time-consuming. Moreover, the deconvolution algorithms require a controlling factor alpha, and the deconvolution algorithms might be unstable if the factor is not chosen properly. Talaat et al. (2020) utilized a chirp signal with the latencies of different frequency components adjusted according to the traveling wave delays of the basilar membrane and found that the chirp-evoked ABR could achieve statistically higher amplitudes within a shorter time. However, the traveling wave delays of the basilar membrane may be highly individual dependent, and the constructed chirp stimulus was usually much longer than the commonly used click sounds, making the maximum stimulation rate largely limited by the stimulus duration. Hence, it is necessary to develop a practical method that could effectively eliminate the influences of MLR and LLR components to obtain reliable ABR waveform morphologies.

The purpose of this study is to propose a random stimulation rate (RSR) technique with the capability of mitigating the interferences arising from the late evoked response from previous stimulation. The performance of the proposed method in improving the ABR waveform morphologies and signal quality was thoroughly evaluated and compared with that of the commonly used FSR method under various stimulation conditions. It is believed that the outcome of this study may potentially aid adequate improvement of ABR measurements towards more accurate hearing loss assessment.

## Materials and Methods

### Subjects

A total of 10 healthy subjects with ages from 20 to 30 years old were recruited in this study (mean age = 24 ± 2.87). The subjects had no history of outer- or middle-ear problems and had normal hearing function with thresholds of 20 dB hearing level or less for frequencies from 250 to 8,000 Hz in standard audiogram tests. The experimental sessions were conducted in an electromagnetically shielded room to prevent acoustical and electromagnetic interferences. The subjects were instructed to sit in a comfortable position and to be as quiet as possible during the test to minimize artifact interferences. The protocol of this study was approved by the Institutional Review Board of Shenzhen Institutes of Advanced Technology, Chinese Academy of Sciences (SIAT-IRB-190615-H0352).

### Experimental Principles

**Figure 1:**
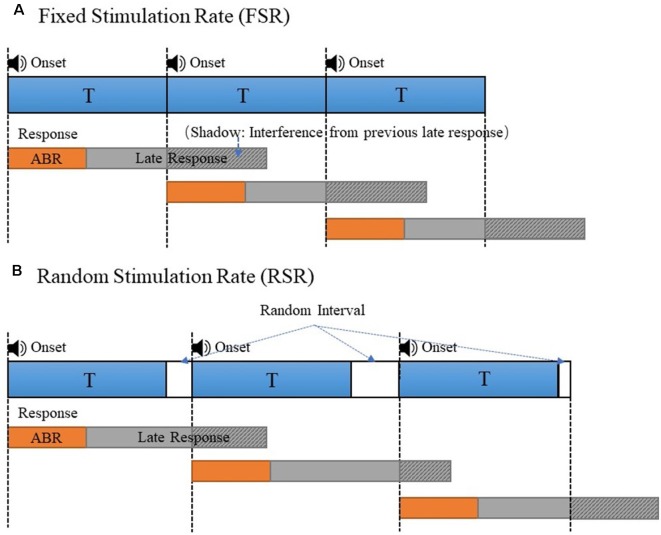
The stimulus presentation comparison of the fixed stimulation rate (FSR; **A**) and random stimulation rate (RSR; **B**) to generate the auditory evoked potential (AEP)s. The overlapped late responses (shadowed area) were synchronous in panel **(A)** and asynchronous in panel **(B)**.

In this study, the method of RSR was proposed to improve the signal quality of ABR measurements. To prevent the ABR signals from being contaminated by interferences from MLR and LLR components of the previous stimulation, the RSR method introduced random time intervals between two adjacent stimulations, as compared with the commonly used FSR method with a fixed stimulus onset interval (Figure 1). As shown in Figure 1, the responses of each stimulation would be averaged in reference to the stimulus onset to obtain the ABR signals, for both the FSR and RSR methods. In Figure 1A, the ABR evoked by the second stimulation overlapped with the late response (MLR and LLR) of the first stimulation, as indicated by the shadow area of the late response. For the FSR method, the overlapped shadowed interferences would be in synchronization with the stimulus onset and therefore be enhanced during the averaging, leading to undesired ABR waveform changes after mixing with the early ABR. In contrast, the shadowed interferences of the RSR method (Figure 1B) no longer synchronized with the stimulus onset after random intervals were introduced, making the shadowed interferences cancel out each other after the averaging. In this way, the interferences of the late responses could be prevented to obtain more accurate ABR results for the proposed RSR method. In Figure 1B, the random interval was set to duration with a uniform distribution between 0 and 10 ms. The stimulation period T was changed from 20 to 70 Hz to systemically investigate the performance of the RSR method under different conditions.

Different stimuli such as clicks, chirps, and tone bursts could be used to evoke ABR signals. The click stimulus is considered the most efficient stimulus for the ABR test due to its easy generation, short duration, and broadband (Eggermont and Moore, 2012; Lu et al., 2017). In this study, the click-based stimulus was adopted for conducting all the ABR tests. The duration of the click stimulus was set to 100 μs throughout the experiments. The earphones that were used to play the click sound were ER-2A (Etymotic Research, Inc., Elk Grove Village, IL, USA), in which plastic tubes were used to connect the earphones and the inserted earplugs to minimize the electromagnetic interferences picked up by the electrodes.

### Experimental Procedures

**Figure 2:**
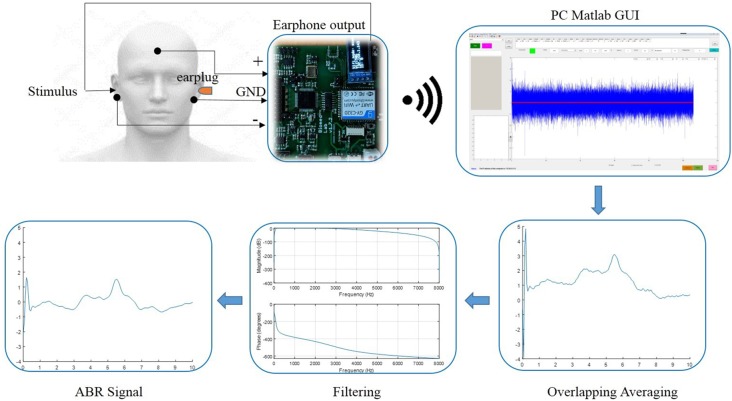
The diagram of the data acquisition system and the configuration of the electrode placement for the auditory brainstem response (ABR) measurements.

The diagram of the system configuration for the ABR data collection was illustrated in Figure 2. A custom wireless hardware platform was built for high-precision ABR measurements. The hardware platform was made up of a low-noise analog frontend, a high-precision analog-to-digital converter (ADC), and the CC3200 Wi-Fi MCU module. Since the ABR amplitude is rather low, the original analog signal was first amplified by an INA188 instrumentation amplifier (Texas Instruments, Dallas, TX, USA) with a gain of 10 and then processed by a high-pass filter with a cutoff frequency of 100 Hz. The filtered signal was then amplified by an INA141 instrumentation amplifier (Texas Instruments, Dallas, TX, USA) with a fixed gain of 100 and finally amplified by a programmable gain amplifier integrated in ADS1299 (Texas Instruments, Dallas, TX, USA), with a gain of 24. The ADS1299 is an ultra-low-noise, 24-bit simultaneous sampling analog-to-digital converter (ADC) that incorporates all commonly required features for extracranial electroencephalogram (EEG) applications. The amplified analog signal was digitized in the ADS1299 analog frontend at a sampling rate of 16,000 Hz, and the raw data were sent from the CC3200 MCU to the PC through Wi-Fi transmission by Transmission Control Protocol (TCP) packets. The electromagnetic interferences introduced by the Wi-Fi transmission were minimized by the randomization of the TCP packet length, so that the interferences of different Wi-Fi TCP packets would not be synchronized to the stimulus onset, leading to cancelation of each other during the averaging. The performance of the custom ABR data collection platform had been evaluated prior to this study, and the internal noise characteristics were comparable to the commercial SynAmps EEG system (Neuroscan). The wireless raw data were then received by a custom MATLAB (MathWorks, Natick, MA, USA) GUI software platform that was capable of real-time digital filtering, ABR waveform averaging, and noise rejection. The raw data were also stored for further off-line analyses.

During the experiments, the subjects were told to comfortably sit on an adjustable backrest chair inside an electromagnetically shielded room. Then the three skin regions mapped out for electrode placement were wiped with alcohol pads: the left mastoid, the right mastoid, and the forehead (Figure 2). Afterward, the reference (inverting) electrodes were placed over the right mastoid, and the active (non-inverting) electrode was placed over the forehead. Finally, the ground electrode was placed over the left mastoid to minimize the common mode of the reference and active electrodes, using the right leg drive technology incorporated in the ADS1229 chip. The impedance between the skin and the electrodes was screened prior to the experiments to ensure it was below 5 kΩ. The impedance difference between the active and reference electrodes was kept below 1 kΩ for satisfactory common-mode rejection. All the electrodes used in this study were disposable snap electrodes with built-in soft gel.

After all the electrodes were in place, the stimulus was generated from the PC and delivered to the ER-2A earphone that was inserted to the right ear. To eliminate the influence of environmental artifacts on the recorded signals, a foam earplug was inserted into the left ear so that the non-test ear would not have impacts on the ABR results. The earphone wires were kept away from the electrode wires as far as possible to avoid possible electromagnetic interferences when playing the stimuli. For each stimulus condition, the stimulation was repeated 2,000 times for both the FSR and RSR methods. Then the responses of the repeated measures were averaged in reference to the stimulus onset so that the synchronous ABR component could be enhanced while the irrelevant noises would be canceled during the averaging.

In this study, each subject participated in four different experimental sessions to systemically evaluate the performance of the proposed RSR method. A rest time of about 5 min was introduced between two consecutive sessions to prevent the subject from possible fatigue which may degrade the signal quality. In session 1, five trials of the same ABR tests evoked by the RSR method were conducted repeatedly, to examine the test–retest reliability of the proposed method. In this session, a stimulation rate of 20 Hz that was close to the commonly adopted settings in the clinic was used, and the sound intensity of the click stimulus was set to 60 dB SPL. In session 2, the stimulation rate was increased from 20 to 70 Hz with an increment of 10 Hz to explore the performance difference between the FSR and RSR methods under different testing speeds. The stimulus sound intensity was fixed at 60 dB SPL for this session. In session 3, the level of the click stimulus was increased from 50 to 65 dB with a step of 5 dB, and ABR signals were measured for both methods. The stimulation rate was constant at 50 Hz. In session 4, the impact of alternate stimulus polarity was investigated for the FSR and RSR methods, with the stimulation rate set to 50 Hz and the stimulus level fixed at 60 dB.

For the data analyses, a digital band-pass filter with cutoff frequencies of 100 and 3,000 Hz was also applied to the raw data to attenuate the out-of-band noises. For the filtered data, an amplitude of 100 μV or greater would be considered as noises (possibly caused by body movements), and the response of the corresponding stimulation would be excluded from the averaging. Then the waveform morphologies and wave I–V latencies of the ABR signals were systemically compared between the FSR and RSR methods, grouped by different stimulus conditions.

## Experimental Results

### Test–Retest Reliability of the RSR Method

**Figure 3:**
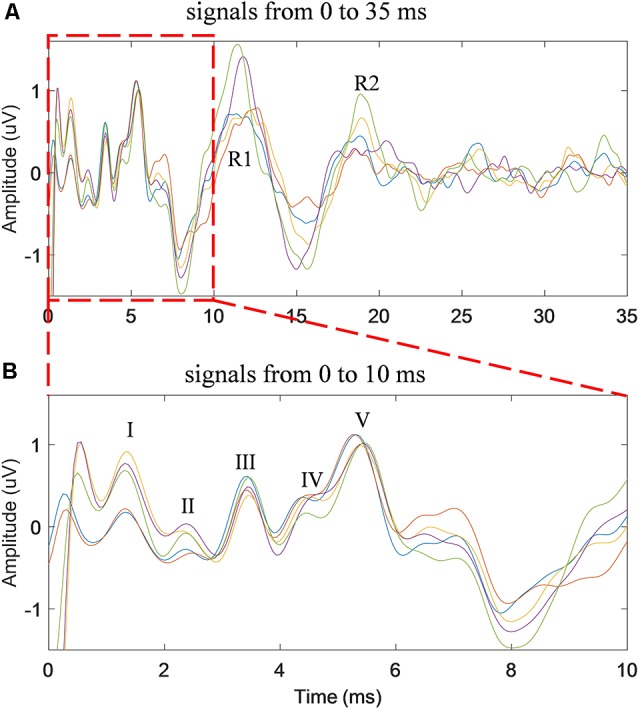
The time waveforms of five repeated trials of the same stimulus condition (stimulation rate of 20 Hz and stimulus level of 60 dB SPL) for the duration of 35 ms **(A)** and 10 ms **(B)** from the stimulus onset.

**Figure 4:**
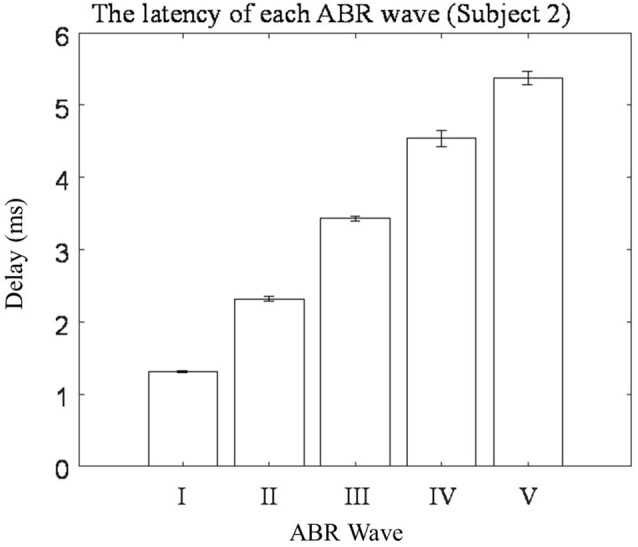
The means and standard deviations of the latencies of wave I–V averaged across all the five repeated trials of ABR tests.

Five repeated trials of the same RSR–ABR measurements were carried out on the same subject, and the temporal waveforms of different trials were compared in Figure 3 in different colors. The stimulation rate was set to 20 Hz, and the stimulus level was 60 dB SPL. It could be observed from Figure 3A, which showed the first 35 ms from the stimulus onset (the beginning of the click stimulus by the earphone), that the peaks and troughs of the five repeated measurements demonstrated good test–retest reliability. It should also be noted that there was a large amplitude of the late responses (such as the peaks of R1 and R2) after the first 10 ms, which could affect the ABR signal if they overlapped with the successive stimulation. The first 10 ms (the actual RSR–ABR signals) of Figure 3A was further examined, and the details were shown in Figure 3B. It could be seen that all standard peaks from wave I to wave V could be clearly identified for the RSR–ABR waveforms, and the morphologies of all the five trials showed great consistency. Then the latency of each peak of the ABR signals in Figure 3 was calculated, and the distribution (mean and standard deviation) of the wave latencies from all the five trials was plotted in Figure 4. It could be observed that the mean latencies of waves I–V were consistent with related reports in subjects with normal hearing (Nazeri et al., 2016; Cargnelutti et al., 2017; Jiang et al., 2019). The maximum standard deviation of the latencies was as low as 0.2 ms, indicating that all the five trials showed rather consistent waveform morphologies. Similar observations could be found from the results of the other subjects.

### Effects of Stimulation Rate

**Figure 5:**
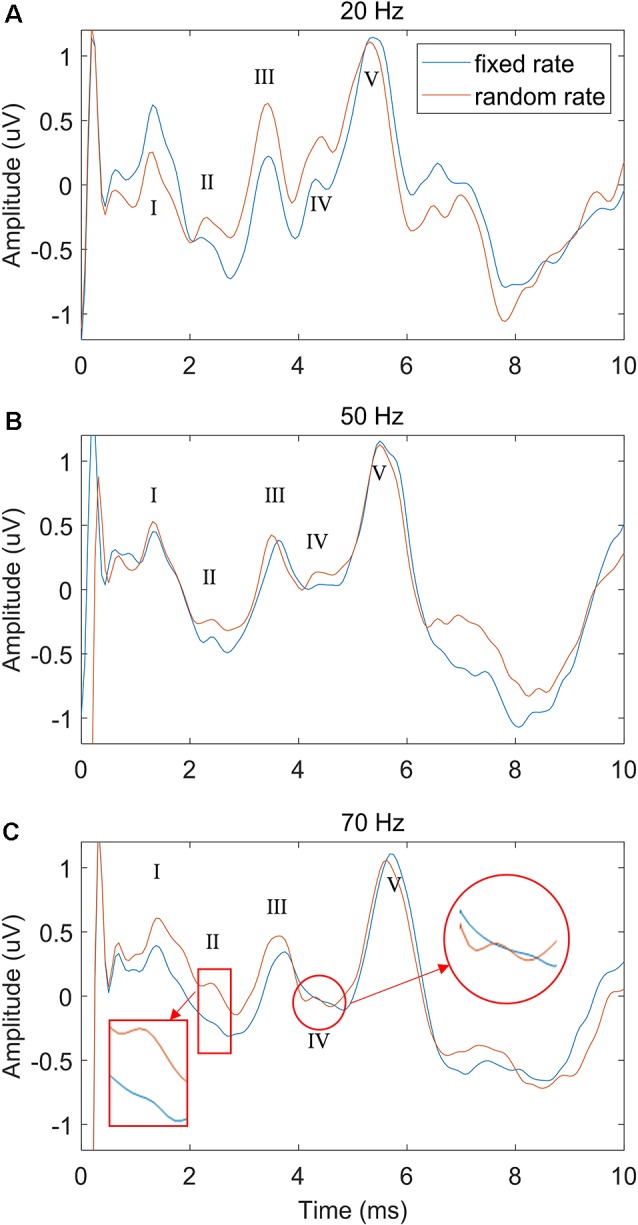
The comparison of ABR waveforms between fixed and random stimulation rates under different repetition speeds of 20 Hz **(A)**, 50 Hz **(B)** and 70 Hz **(C)**, with the stimulation level fixed at 60 dB SPL.

To explore the performance difference between the FSR and RSR methods under different stimulus repetition speeds, the stimulation rate was increased from 20 to 70 Hz, and the comparisons under different speeds (20, 50, and 70 Hz) were shown in Figure 5 (stimulus level = 60 dB SPL). Generally, the overall ABR waveforms of the two methods were rather consistent across different stimulation rates. However, as compared with the FSR method, the RSR method could achieve better ABR morphologies indicated by clearer waveform differentiation, especially at high stimulation rates (Figure 5C). All peaks from waves I to V could be easily recognized for the RSR method regardless of the stimulation rate, whereas waves II and IV were not visible for the FSR method at the stimulation rate of 70 Hz.

### Effects of Stimulus Level

**Figure 6:**
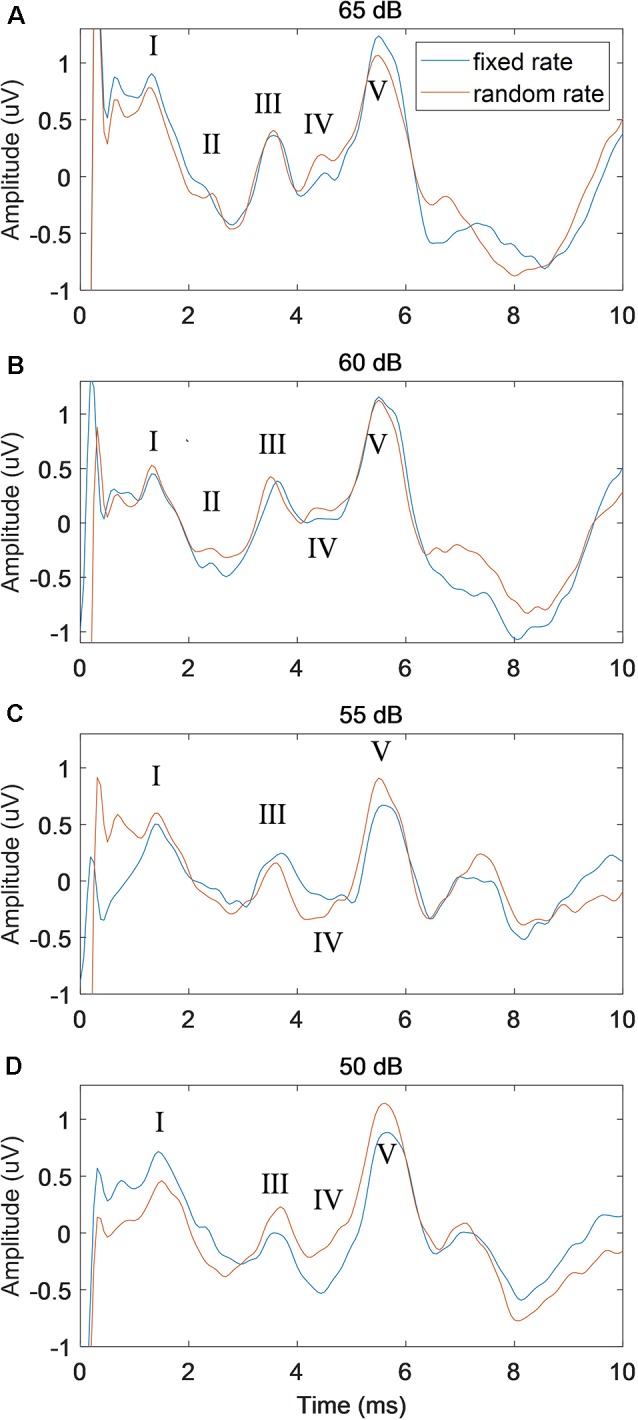
The comparison of ABR waveforms between fixed and random stimulation rates under different stimulus levels of 65 dB **(A)**, 60 dB **(B)**, 55 dB **(C)** and 50 dB **(D)** SPL, with the stimulation rate fixed at 50 Hz.

To further examine the performance of the proposed RSR method, the stimulus level was increased from 50 to 65 dB with a step of 5 dB, and the comparisons with the conventional FSR method were shown in Figure 6 (stimulation rate = 50 Hz). While the waveforms of both methods deteriorated as the stimulus level decreased, the RSR method was less affected by random noises and demonstrated slightly smoother morphologies. The RSR method also showed larger wave V amplitudes at lower stimulus levels of 55 and 50 dB SPL. Similar effects of stimulus level were observed in the results of other subjects.

### Effects of Stimulus Polarity

**Figure 7:**
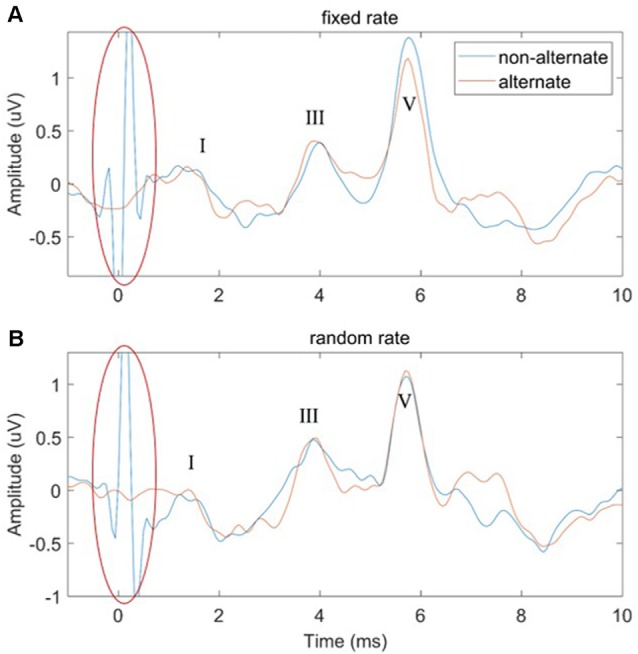
The comparison of ABR waveforms using non-alternate and alternate stimulus polarities for the fixed-rate **(A)** and random-rate **(B)** methods.

In this study, two ways of manipulating the stimulus polarity were performed: non-alternate (only condensation click stimuli were used) and alternate (the condensation and rarefaction clicks were used alternately). The effects of stimulus polarity on the performance of both the FSR and RSR methods were compared in Figure 7. It could be observed that large amplitudes of stimulus artifacts were present at the stimulus onset (*t* = 0 ms, marked by red ovals) for the non-alternate stimulus polarity. In contrast, the alternate stimulus polarity approach could eliminate such stimulus artifacts by canceling the stimulus-related components of the condensation and rarefaction clicks. However, no significant effects of the stimulus polarity on the amplitudes and latencies of waves I to V were observed, for both the FSR and RSR methods.

### Effects of Averaging Times

**Figure 8:**
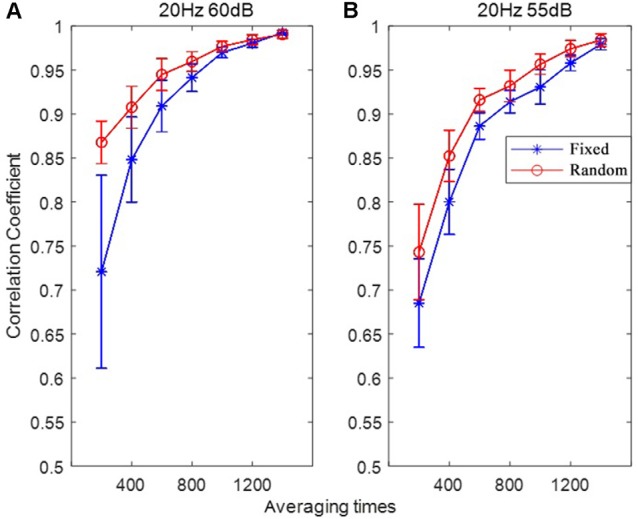
The correlation coefficients between the ABR waveform with varying averaging time and the final ABR waveform (averaged for 2,000 times) under the stimulation conditions of 20 Hz, 60 dB **(A)** and 20 Hz, 55 dB **(B)**.

In order to investigate the speed of convergence (stabilization) of the RSR method, the correlation coefficients between the ABR waveform of increasing average times (from 200 to 1,400 with an increment of 200) and the final ABR waveform (averaged a total of 2,000 times) were calculated for each subject. Then the correlation coefficients of all the subjects were analyzed, with the correlation coefficient statistics (mean and standard deviation) plotted as functions of averaging times (or repeated times) shown in Figure 8. The most noteworthy observation was that the convergence speed of the RSR method was significantly faster than that of the conventional FSR method for different stimulus levels, indicating that the proposed RSR method could achieve ABR waveforms similar to the final results (averaged 2,000 times) much earlier. For the same averaging time, the correlation coefficient of the RSR method was also significantly higher. Especially at the stimulus level of 60 dB, the mean correlation coefficient of the RSR method was as high as 0.87 for the averaging time of only 200, whereas the mean correlation coefficient of the FSR method was only 0.72 as a comparison.

## Discussion

Although some studies were made on the medical system platform and acquisition system (Han et al., 2019; Pirbhulal et al., 2019; Sun et al., 2020), it helped less with the improvement of ABR morphology. Towards effectively solving the problem on the quality of ABR signals, the RSR method was proposed to improve the morphology and reliability of current ABR measurements. By integrating a random interval between two adjacent stimulations, the proposed method could mitigate the interferences arising from the late evoked response of the previous stimulation. The performance of the proposed method was systemically evaluated and compared with the currently used FSR method under various stimulation conditions using a custom wireless high-precision data acquisition platform.

### Effects of Stimulation Rate

The stimulation rate has been proven as an important factor that significantly affects the ABR results in the context of clinical hearing loss diagnosis (Schwartz and Morris, 1991; Musiek et al., 1994). In this study, different stimulation rates from 20 to 70 Hz were used to evoke the ABR potentials. The results showed that the ABR waveform differentiations deteriorated as the stimulation rate increased (Figure 5), and the findings are consistent with the other studies (Don et al., 1977; Kjær, 1980; Lasky, 1984; Valderrama et al., 2012). Moreover, it was found that at high stimulation rates such as 70 Hz, waves II and IV started to disappear for the conventional FSR method (Alvarez et al., 2010; Valderrama et al., 2012). The reason might be that a large amplitude of late response (10 ms after the stimulus onset, as seen in Figure 3A) overlapped with the response of the successive stimulation, given that there was only about 14 ms of time gap between the two stimulus onsets. The previous overlapped late response mixed with the ABR signal of the current stimulation and would be synchronously enhanced after the averaging for the FSR method (Figure 1A), leading to significant morphology changes in the ABR measurements. As a comparison, the proposed RSR method could eliminate the synchrony of the overlapped late responses, making them cancel each other during the averaging. Therefore, the waveform differentiation of the RSR method was significantly improved when compared with the FSR method, indicated by the presence of waves II and IV even at a stimulation rate of 70 Hz (Figure 5C). The proposed method could make the ABR measurements to be carried out with a faster stimulation rate while maintaining reliable waveform differentiations.

### Effects of Stimulus Level

A general observation on the effects of the stimulus level was that better ABR waveform morphologies and shorter wave V latencies could be seen at higher stimulus levels (Figure 6), which is consistent with other studies (Serpanos et al., 1997; Louza et al., 2016; Rouillon et al., 2016). Figure 6 also showed that the proposed RSR method could obtain cleaner ABR signals and better waveform morphologies when compared with the FSR method. The finding might be explained by the cancellation of the overlapped late response of the previous stimulation, as well as other noise sources that were synchronized with the stimulus onset. For low stimulus levels such as 55 and 50 dB, the RSR method could also obtain a larger amplitude of wave V (Figures 6C,D), indicating that the proposed RSR method might be able to measure ABR thresholds at lower stimulus levels and therefore could provide more accurate results for clinical evaluation of hearing functions.

### Effects of Stimulus Polarity

In this study, Figure 7 showed that the large amplitude of stimulus artifacts happening at the stimulus onset could be efficiently eliminated by alternatively changing the polarity of the click stimuli. Similar findings are also reported by other studies when altering the stimulus polarity to measures ABR signals (Gorga et al., 1985; Akhoun et al., 2008; Hornickel et al., 2012; Anderson et al., 2013; Ahadi et al., 2014; Mamo et al., 2016). This can be explained by the linear relationship between the stimulus artifacts and the click polarity. However, no significant effects of the stimulus polarity on the amplitudes and latencies of the waveform peaks were observed for both the FSR and RSR methods. Salt and Thornton (1984) also found that the major component of the ABR was insensitive to stimulus polarity. Regarding the latency of wave V, while some studies reported that there were some differences when changing the stimulus polarity (Borg and Löfqvist, 1981; Hughes et al., 1981; Pijl, 1987), other studies showed that there was no significant difference at all (Rosenhamer et al., 1978; Beattie and Boyd, 1984; Tietze and Pantev, 1986; Kumar et al., 2014). The discrepancy might be attributed to the high sensitivity of ABR signals to various noises. The present study suggests that stimulus polarity is not an important factor when choosing the stimuli in routine clinical ABR measurements.

### Effects of Averaging Times

Another important finding of this study is that the convergence speed of the RSR method was significantly faster for different stimulus levels when plotting the correlation coefficients as a function of averaging time (Figure 8), indicating that the proposed method could obtain stable ABR waveform earlier than the conventional FSR method. With an averaging time of only 200, the mean correlation coefficient between the current and final ABR waveforms was as high as 0.87, given that over 4,000 averages are usually required to achieve a satisfactory result (Johnson et al., 2008; Hornickel et al., 2009; Skoe and Kraus, 2010; Skoe et al., 2015). The statistics in Figure 8 suggest that the proposed RSR method is superior in preventing undesired noises from contaminating the target ABR signals so that the waveforms could be stabilized faster than the currently used FSR method. Therefore, the efficiency of current ABR tests could be significantly improved by incorporating the RSR paradigm into clinical settings.

### Clinical Implications

In clinical applications, ABR parameters such as the amplitude of peaks, the absolute latency of waves I–V, interpeak latency, and interaural latency are of great importance for the diagnoses of hearing loss and other hearing impairments. However, all these ABR parameters heavily rely on the ABR signal quality and waveform morphologies. Given that the ABR waveform of the conventional fixed-rate method might be affected by interferences originating from the previous late response, the proposed RSR showed great performance in improving the ABR waveform differentiation under different stimulus conditions. Generally, the amplitude and latency of waves I, III, and V and their inter-wave latencies will be taken as the diagnostic parameters for hearing impairments. However, the proposed RSR method could help to identify clear waves II and IV, whose amplitudes and latencies also provide rather useful information for clinical diagnoses. For example, the inter-wave latency of waves III and IV reflects axonal conduction time, and the interval of wave IV and V reflects a synaptic delay (De Vries and Glass, 2019). Lee et al. (2018) reported that the amplitude of wave II could be considered a supplementary indicator to help with the diagnosis of vestibular paroxysmia.

Compared with Valderrama’s iterative-RSA method that might involve complex deconvolution algorithms and empirically chosen controlling factors (Valderrama et al., 2014), the implementation strategy of the proposed method is much simpler, with only minimal changes to the stimulus presentation of the current commercial systems. The easy implementation of the proposed method would help to greatly reduce the cost while achieving significant improvement in ABR signal qualities, making it rather useful in medical application scenarios such as intraoperative monitoring during surgery, auditory threshold estimation, and newborn hearing screening.

## Conclusion

In this study, an RSR method was proposed, and the performance on improving the morphology and reliability of ABR signals was systemically investigated under different stimulus conditions. The results showed that the RSR method demonstrated great test–retest reliability in repeated measurements. By canceling the interferences of the late response from the previous stimulation, it could also achieve better ABR morphologies indicated by clearer waveform differentiation under different stimulation rates and stimulus levels. The RSR method could obtain satisfactory results significantly faster than the conventional FSR method, and it could help to greatly improve the efficiency of current ABR measurements. The proposed RSR method may provide a candidate tool that would aid accurate and efficient diagnoses of hearing impairment in clinical settings. The approach of obtaining reliably evoked potentials from the brain might also be helpful for applications such as brain–computer interface and intelligent control of robotic systems.

## Data Availability Statement

The datasets generated for this study are available on request to the corresponding author.

## Ethics Statement

The protocol of this study was approved by the Institutional Review Board of Shenzhen Institutes of Advanced Technology, Chinese Academy of Sciences (SIAT-IRB-190615-H0352). The participants provided their written informed consent to participate in this study.

## Author Contributions

XinW, MZ, and SC contributed to the conception and design of the study. OS, XiaW, and HZ conducted experiments. YL and WW analyzed the data. SM contributed to the choice of the electrodes. XinW wrote the first draft of the manuscript. JY, MW, and GL contributed to manuscript revision. All authors read and approved the submitted manuscript.

## Conflict of Interest

The authors declare that the research was conducted in the absence of any commercial or financial relationships that could be construed as a potential conflict of interest.
